# Adsorption mechanisms of atrazine isolated and mixed with glyphosate formulations in soil

**DOI:** 10.1371/journal.pone.0242350

**Published:** 2020-11-25

**Authors:** Matheus de Freitas Souza, Ana Claudia Langaro, Ana Beatriz Rocha de Jesus Passos, Hamurábi Anizio Lins, Tatiane Severo Silva, Vander Mendonça, Antônio Alberto da Silva, Daniel Valadão Silva

**Affiliations:** 1 Department of Agronomic, Universidade Federal Rural do Semi-Árido, Mossoró, Rio Grande do Norte, Brazil; 2 Department of Crop Production, Universidade Federal de Viçosa, Viçosa, Minas Gerais, Brazil; Qatar University, QATAR

## Abstract

In Brazil, the atrazine has been applied frequently to join with glyphosate to control resistant biotypes and weed tolerant species to glyphosate. However, there are no studies about atrazine's behavior in soil when applied in admixture with glyphosate. Knowledge of atrazine's sorption and desorption mixed with glyphosate is necessary because the lower sorption and higher desorption may increase the leaching and runoff of pesticides, reaching groundwaters and rivers. Thereby, the objective of this study was to evaluate the adsorption mechanisms of atrazine when isolated and mixed with glyphosate formulations in a Red-Yellow Latosol. The maximum adsorbed amount of atrazine in equilibrium (*q*_*e*_) was not altered due to glyphosate formulations. The time to reach equilibrium was shortest when atrazine was mixed with the Roundup Ready^®^ (*te* = 4.3 hours) due to the higher adsorption velocity (*k*_*2*_ = 2.3 mg min^-1^) in the soil. The highest sorption of atrazine occurred when mixed with the Roundup WG^®^, with the Freundlich sorption coefficient (*Kf*) equal to 2.51 and 2.43 for both formulation concentrations. However, other glyphosate formulations did not affect the sorption of atrazine. The desorption of atrazine was high for all treatments, with values close to 80% of the initial adsorbed amount, without differences among isolated and mixed treatments. The change in the velocity and capacity of sorption for the atrazine mixed with some glyphosate formulations indicates that further studies should be conducted to identify the mechanisms involved in this process.

## Introduction

The application of pesticides is a common practice among producers and aims to ensure the maximum productivity of crops. Among the pesticides (herbicides, insecticides, and fungicides), herbicides occupy the top of the ranking, accounting for 65% of the total marketed [1]. According to this same study, glyphosate is the best-selling active ingredient in the country.

The intense use of glyphosate increased the selection pressure on resistant biotypes and tolerant weeds to this herbicide, increasing the number of crops infested with these species [2]. In Brazil, eight species have been identified as resistant to glyphosate [3], and the rapid dissemination has raised the control costs. Among the tools to control the resistant or tolerate weeds to the glyphosate, the application of two or more herbicide molecules with different mechanisms of action has been shown to be effective in reducing resistant populations [4]. These applications are usually carried out in sequence or by mixing the molecules in a single application. One of the herbicides that have been used in mixture with glyphosate is atrazine. This herbicide is selective for maize crop and efficient to control species such as *Conyza bonariensis*, *Commelina bengalensis*, and *Commelina difusa* [5].

When an herbicide is applied, it is estimated that 70% reach the soil [5]. Thereby, the interactions between herbicides and soil should be understood to avoid environmental contamination. Although glyphosate + atrazine application is common among producers in Brazil, our knowledge is not sufficient about the behavior of these mixed pesticides to reach the soil. In some situations, herbicides can migrate to different compartments and contaminate the soil and water resources [6]. Among herbicides, those with high mobility and persistence has higher risk of contaminating natural resources, such as groundwater and surface water through leaching and surface runoff, respectively [7].

Although studies bring solutions for remediation of areas contaminated by atrazine [8, 9], to avoid the environmental pollution by this herbicide is the main way for a sustainable agriculture. Thus, studies to understand the interactions between herbicide and soil must be conducted to avoid the environmental pollution by this herbicide is the leading way for sustainable agriculture. The mobility and persistence of an herbicide in the soil depend on the interactions between the molecule and soil organic and mineral fractions. Among these interactions, sorption and desorption define the availability of herbicide in the soil solution, and once in this fraction, herbicide can leach and contaminate water resources [10].

Atrazine (2-chloro-4-ethylamino-6-isopropylamino-s-triazine) is a herbicide belonging to the group of triazines, with a melting point of 175°C, and water solubility of 33 mg L^-1^, being considered as a weak base (pKa = 1.7). Studies have already reported the presence of this herbicide in water sources, such as rivers and groundwater, and researchers have already characterized the mobility of atrazine applied alone in different soils [11, 12]. Studies have shown that atrazine can establish electrostatic and Van der Waals bonds with organic minerals and soil minerals capable of reducing the mobility of this herbicide [13]. The adsorption via host-guest is also a mechanism already reported to be responsible for adsorbing the atrazine in aqueous solutions rice husk functionalized with β-cyclodextrin [14]. However, these studies just evaluated the isolated applications of this herbicide. If we consider that the presence of another pesticide molecule as a factor able to alter herbicide behavior, it is necessary also to investigate the retention processes of herbicides in associated applications. [15] studying the impact of the mixture of chemical compounds, reported that the mixture between methyl tert-butyl ether and tert-butyl alcohol altered the adsorption processes. Moreover, the H_2_PO_4_^+^ aqueous solution reduced the adsorption of glyphosate in different biochars used for water decontamination [16].

We hypothesize in this work that the presence of glyphosate formulations in associated applications can influence the adsorption processes of the atrazine in the soil. We believe that glyphosate may compete for soil sorption sites, manly electrostatic interactions, preventing atrazine sorption. Also, we consider that the presence of other inert components may affect atrazine sorption and desorption. Therefore, our objective was to evaluate, through the chromatographic method, the sorption and desorption of atrazine isolated and mixed with formulations of glyphosate. The novelty of these results may demonstrate that a practice often used does not increase the risk of environmental contamination.

## Material and methods

### Sample collection and preparation

The experiment was carried out at the Soil Herbicide Laboratory of the Universidade Federal de Viçosa, Brazil. Samples of a Red-Yellow Latosol were collected at the coordinates: latitude 20° 44′ 37.8″ and longitude 42° 50′ 40″ W, at an altitude of 650 m. The samples were collected, dried, and sieved in a four mesh, after which the physical and chemical analysis was carried out (Table 1).

**Table 1:** Results of the physical, chemical, and textural analysis of samples of the Red-Yellow Latosol used in this work.

Soil	pH (H_2_O)	P	K	Ca^2+^	Mg^2+^	Al^3+^	H+Al	(t)	Organic matter
		(mg kg^-1^)			(cmol_c_ kg^-1^)					%
	4.7	2.33	41	2.2	0.7	0.2	5.61	3.20	2.52
Soil	Sand	Silt				Clay			Texture class
	%								
	71	7				17			Franco-sandy

### Determination of atrazine

The determination of atrazine was carried out by liquid chromatography (2-chloro-4-ethylamino-6-isopropylamino-s-triazine) obtained from a 1.000 mg L^-1^ stock solution of atrazine (analytical standards) in methanol on the substrate. The equipment used was a Shimadzu^®^ LC 20AT chromatograph, chromatograph, with diode split detector (Shimadzu SPD 20A) and stainless steel column C18 (Shimadzu VP-ODS Shim-pack 280 mm x 4.6 mm d.i. x 5 μm particle diameter). The chromatographic conditions for the analysis were: mobile phase composed of distilled water and methanol in the proportion of 40:60 (v/v), a flow of 1.0 mL min^-1^, the injection volume of 20 μL, a temperature of 45°C and wavelength of 221 nm. The retention time of atrazine under these conditions was approximately 7.50 minutes. Analytical curve parameters estimated the concentration. The identification was made by the retention time using an atrazine analytical standard.

### Preparation of glyphosate formulations

Stock solutions 1.000 mg L^-1^ of four formulations of glyphosate, Roundup WG^®^, Roundup Ready^®^, Roundup Ultra^®^, and Zapp Qi^®^ were prepared in distilled water and stored in a refrigerator. From these stock solutions, the concentrations, 10 and 50 mg L^-1^ were obtained for each formulation, used in the equilibrium and sorption time tests. These concentrations were based on the glyphosate doses (720 and 1440 kg ha^-1^ a.i) used to control weeds.

### Adsorption kinetics

The determination of the time required to equilibrate the herbicide concentration adsorbed and in the soil solution, and the sorption and desorption of the atrazine alone and mixed with glyphosate formulations were carried out according to Organization for Economic Co-operation and Development (OECD) recommendations [17].

A solution of 0.01 mol L^-1^ CaCl_2_ containing 10 mg L^-1^ of atrazine was prepared as a stock solution. From this solution, 10.0 mL was added in polypropylene tubes containing 2.00 g of the substrate to evaluate the equilibrium time of the isolated atrazine. In the treatments contained atrazine plus glyphosate formulations, 2.00 g of the substrate were added in the tubes with 5.00 mL of a solution of 20 mg L^-1^ atrazine and 5.00 mL of a solution of glyphosate at the concentration of 10 or 50 mg L^-1^ (varying according to each treatment).

Subsequently, the tubes were shaken vertically at different times at a speed of 50 rpm (0.5; 1.0; 2.0; 3.0; 4.0; 8.0; 12; 16 and 24 hours), the temperature of 27 ± 2°C. After stirring, the samples were centrifuged at 3.500 rpm for six minutes. From the supernatant, 2 mL was removed and filtered in a Millipore filter with 0.45 μm polytetrafluoroethylene membrane (PTFE) for chromatographic analysis. The equilibrium time chosen was determined from which the concentration of the solution remained constant. Two models, pseudo-first (Eq 1) order and pseudo-second order (Eq 2) were utilized to describe the atrazine adsorption kinetic.

$$q_{\left(t\right)}=q_{e}[1-\exp\left(-k_{1}*t\right)]$$ Eq (1)

$$q_{\left(t\right)}=q_{e}*\frac{k_{2}*t}{1+k_{2}*t}$$ Eq (2)

where *q*_*(t)*_: quantity adsorbed in time; *q*_*e*_: amount adsorbed at equilibrium; *t*: time; *k*_*1*_: constant of pseudo-first order; *k*_*2*_: constant of pseudo-second order.

### Sorption isotherms

The isolated atrazine sorption was carried out from working solutions at concentrations of 0.4; 0.75; 1.25; 2.5; 5.0; and 10.0 mg L^-1^ in 0.01 mol L^-1^ CaCl_2_, 10.0 mL of these solutions being added in polypropylene tubes containing 2.00 g of the substrate. The sorption of atrazine mixed with glyphosate formulations was performed from working solutions at concentrations of 0.8; 1.5; 2.5; 5.0; 10.0; and 20.0 mg L^-1^ in 0.01 mol L^-1^ CaCl_2_ of atrazine. In tubes were added: 2.0 g of the substrate, 5.00 mL of working solutions, and 5.0 mL of glyphosate in the concentration of 10 or 50 mg L^-1^ calculated considering each formulation (WG^®^, Roundup Ready^®^, Ultra^®^ or ZAPP Qi^®^).

Subsequently, these tubes were shaken vertically at a temperature of 27 ± 2°C and 50 rpm, for the equilibrium time determined in the previous step. After stirring, the samples were centrifuged at 2260 x g for six minutes. The supernatant was removed and filtered in a 0.45 μm Millipore filter for chromatographic analysis.

Concentrations of atrazine sorbed to the substrate (*C*_*s*_) in mg kg^-1^ were calculated by the difference between the concentration initially added to the soil and the amount found after the equilibrium (*C*_*e*_). From the values of *C*_*e*_ and *C*_*s*_, the Freundlich (Eq 3) and Langmuir (Eq 4) were adjusted to give the sorption coefficients, where *K*_*f*_ and *1/n* are empirical constants representing the capacity and sorption intensity, respectively.

$$C_{s}=k_{f}*{C_{e}}^{1/n}$$ Eq (3)

$$Q_{t}=\frac{q_{max}*kl*C_{e}}{1+kl*C_{e}}$$ Eq (4)

where *C*_*s*_: concentration adsorbed mg kg^-1^; *K*_*f*_: sorption coefficient for the Freundlich model μmol kg^-1^; *1/n*: linearity index of Freundlich; *Q*_*t*_: amount sorbed mg kg^-1^; *q*_*max*_: maximum adsorption capacity; *Kl*: Langmuir model constant; *C*_*e*_: quantity in the soil solution mg L^-1^.

### Desorption

In the desorption test, 5,0 mL of CaCl_2_ 0.01 mol L^-1^ herbicide-free solution was added to the same tubes to promote the shift in the balance and observe the reversibility of the sorption process. These tubes were subjected to a new agitation during the same period and temperature of the sorption tests. After the agitation, the samples were centrifuged at 3.500 rpm, for six minutes. After this procedure, 5.0 mL of the supernatant were removed and filtered in membrane of 0.45 μm for chromatographic analysis.

The amount of herbicide still sorbed in the soils at each desorption stage was calculated by the difference between the amount of the herbicide sorbed and amount desorbed. The desorption was calculated as percentage (%) in relation to the atrazine amount sorbed initially.

### Statistical analysis

The kinetics and isotherms models were tested by the Akaike test, root mean square error (RMSE), and coefficient of determination (*R*^*2*^) to determine the best model [19]. All the data were submitted to the variance analysis by test F. When significant, the means were compared by Tukey to the p-value ≤ 0.05, and the differences show as tables or figures.

$$RMSE=\sqrt{\frac{1}{N-2}\sum_{i=1}^{N}{\left(q_{e}(exp)-q_{e}(calc)\right)}^{2}}$$ Eq (5)

$$R^{2}=1-\frac{\sum_{i=1}^{N}{\left(y_{1-}y_{2}\right)}^{2}}{\sum_{i=1}^{N}{{(y}_{1-}(y))}^{2}}$$ Eq (6)

where *q*_*e*_*(exp)* and *q*_*e*_*(calc)* represent the experimental and calculated values of the adsorption capacity (mg g^-1^) and N is the number of experimental data. In which *(y)* stands for the average value of the *y*_*1*_*’s* (*i* = 1,…, *N*), that is *(y)* = (1*/N*) $\sum_{i=1}^{N}y1$. The lowest values of *R*^*2*^ and *RMSE* indicate the best model fitting and the similarity of model with the experimental data, respectively.

**Fig 1:**
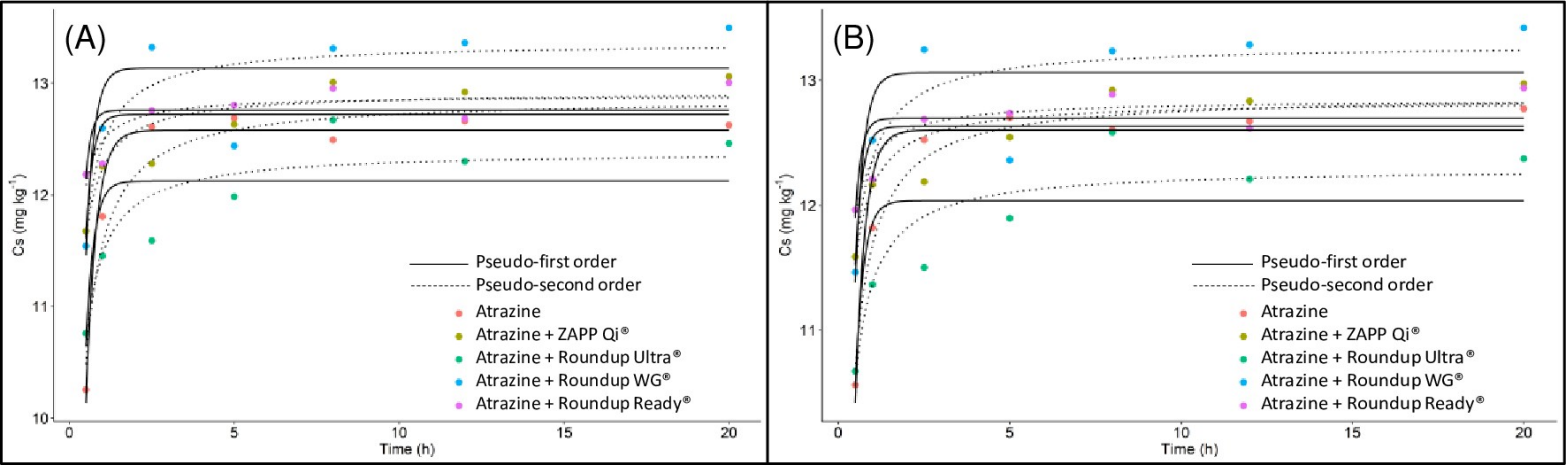
Sorption kinetic curve in the atrazine Red-Yellow Latosol isolated and mixed with glyphosate formulations at concentrations of 10 mg L^-1^ (A) and 50 mg L^-1^ of acid equivalent (B).

**Fig 2:**
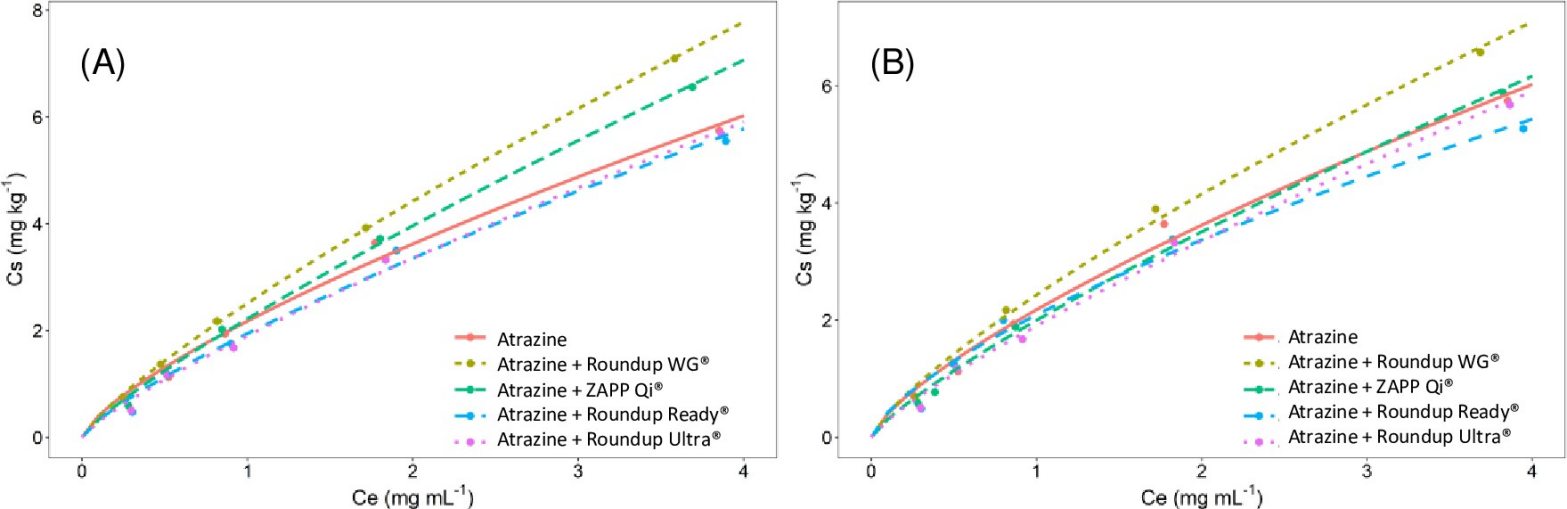
Freundlich isotherms for sorption, in a Red-Yellow Latosol, of atrazine isolated and mixed with glyphosate formulations at 10 mg L^-1^ (A) and 50 mg L^-1^ of acid equivalent (B).

The linearity of the method, analytical curve, selectivity, and detection and quantification limits were evaluated by analyzing the supernatants resulting from the stirring of the soil with 10 mL of 0.01 mol L^-1^ CaCl_2_ solution without and with atrazine (Fig 1). Linearity was determined using five triplicate concentrations of 0.1 to 10 mg L^-1^ in 0.01 mol L^-1^ CaCl_2_ (Fig 2). The limit of detection (LOD) and limit of quantification (LOQ) for atrazine isolated and mixed with glyphosate formulations were determined as shown in Table 2.

**Table 2:** Kinetic adsorption models of atrazine isolated and mixed with glyphosate formulations (10 mg L^-1^) in a Red-Yellow Latosol.

Treatment	Pseudo-first order				Pseudo-second order			
	Parameters	Estimated	Std. Error	R^2^	Parameters	Estimated	Std. Error	R^2^
Atrazine	*q*_*e*_	12.6	0.07***	0.97	*q*_*e*_	12.9	0.12***	0.99
	*k*_*1*_	3.3	0.14***		*k*_*1*_	0.7	0.09***	
Atrazine + ZAPP Qi^®^	*q*_*e*_	12.7	0.14***	0.97	*q*_*e*_	12.9	0.11***	0.99
	*k*_*1*_	4.9	0.67***		*k*_*1*_	1.4	0.28**	
Atrazine + Roundup Ready^®^	*q*_*e*_	12.8	0.10***	0.99	*q*_*e*_	12.9	0.07***	0.99
	*k*_*1*_	6.1	0.88***		*k*_*1*_	2.3	0.52**	
Atrazine + Roundup WG^®^	*q*_*e*_	13.1	0.18***	0.97	*q*_*e*_	13.4	0.19***	0.99
	*k*_*1*_	4.1	0.52***		*k*_*1*_	1.0	0.29*	
Atrazine + Roundup Ultra^®^	*q*_*e*_	12.1	0.19***	0.98	*q*_*e*_	12.4	0.14***	0.99
	*k*_*1*_	4.2	0.64**		*k*_*1*_	1.0	0.21**	

***Significant to p-value ≤ 0.001.

**Significant to p-value ≤ 0.01.

*Significant to p-value ≤ 0.05. *q*_*e*_ *=* Concentration adsorbed at equilibrium (mg kg^-1^). *k*_*1*_ = first-order constant (min^-1^). *k*_*2*_ = second-order constant (mg min^-1^).

## Results and discussion

### Adsorption kinetics

The kinetic models of the pseudo-first order and pseudo-second order were used to describe the behavior of atrazine isolated and mixed with glyphosate formulations at concentrations of 10 mg L^-1^ and 50 mg L^-1^ (Fig 1A and 1B). Both models had low standard error values for the parameters, varying 5% for Pseudo-First Order (PFO) and Pseudo-Second Order (PSO) (Tables 2 and 3). Also, all parameters were significant (p-value) for treatments with atrazine applied alone and in combination with glyphosate at the concentrations of 10 mg L^-1^ (Table 2) and 50 mg L^-1^ (Table 3). This result indicates that the two models can describe the behavior of atrazine when submitted to the treatments applied to the soil. However, choosing a more data-fit model ensures greater precision to estimate some critical values, such as velocity and concentration in equilibrium [18, 19].

**Table 3:** Kinetic adsorption models of atrazine isolated and mixed with glyphosate formulations (50 mg L^-1^) in a Red-Yellow Latosol.

Treatment	Pseudo-first order				Pseudo-second order			
	Parameters	Estimated	Std. Error	R^2^	Parameters	Estimated	Std. Error	R^2^
Atrazine	*q*_*e*_	12.6	0.093***	0.96	*q*_*e*_	12.9	0.07***	0.99
	*k*_*1*_	3.5	0.21***		*k*_*1*_	0.8	0.07***	
Atrazine + ZAPP Qi^®^	*q*_*e*_	12.6	0.14***	0.95	*q*_*e*_	12.8	0.11***	0.99
	*k*_*1*_	4.9	0.67***		*k*_*1*_	1.4	0.28**	
Atrazine + Roundup Ready^®^	*q*_*e*_	12.7	0.10***	0.97	*q*_*e*_	12.8	0.06***	0.99
	*k*_*1*_	5.6	0.67***		*k*_*1*_	2.0	0.33**	
Atrazine + Roundup WG^®^	*q*_*e*_	13.1	0.18***	0.98	*q*_*e*_	13.3	0.20***	0.99
	*k*_*1*_	4.1	0.52***		*k*_*1*_	1.0	0.29*	
Atrazine + Roundup Ultra^®^	*q*_*e*_	12.0	0.19***	0.94	*q*_*e*_	12.3	0.14***	0.99
	*k*_*1*_	4.2	0.64**		*k*_*1*_	1.0	0.21**	

***Significant to p-value ≤ 0.001.

**Significant to p-value ≤ 0.01.

*Significant to p-value ≤ 0.05. *q*_*e*_ *=* Concentration adsorbed at equilibrium (mg kg^-1^). *k*_*1*_ = first-order constant (min^-1^). *k*_*2*_ = second-order constant (mg min^-1^).

The selection of the best model considered the values obtained by the Akaike test (AIC_c_), the root mean square error (RMSE), and coefficient of determination (R^2^) [20]. The PSO model observed values for AIC_c_ and RMSE were 11.60 and 0.10, and 9.60 and 0.09 for 10 and 50 mg L^-1^ of glyphosate, respectively. Similar values were observed for R^2^ (0.98) in all treatments (Table 4). For the PFO model, the values for AIC_c_ and RMSE were 14.80 and 0.12, and 15.60 and 0.13 for 10 and 50 mg L^-1^ of glyphosate, respectively (Table 4). For R^2^, the observed values were equals in all treatments (Table 4). Both criteria, Akaike and RMSE, the lowest observed value indicate that the model presents better adjustment to the data considering each one's number of parameters [20]. Therefore, for the set of data evaluated, the PSO model showed a better fit and should be chosen for a more accurate interpretation of the data. The adjustment of atrazine adsorption data for PSO in RYO indicates a possible chemosorption between the adsorbent and the adsorbate. Chemosorption was also observed for diuron and hexazinone, photosystem II inhibiting herbicides, as well as atrazine, in two Brazilian oxisols [21]. This type of adsorption does not always occur for atrazine, as reported by where the PFO model was better to explain the adsorption of atrazine in an organic adsorbent [14].

**Table 4:** Values of the Akaike test (AICc) and root mean square error (RMSE) for the kinetic models of pseudo first order and pseudo second order, and Freundlich and Langmuir isotherms of atrazine isolated and mixed with glyphosate formulations.

Models	10 mg L^-1^		50 mg L^-1^	
	AIC_c_	RMSE	AIC_c_	RMSE
Pseudo-second order	11.60	0.10	9.60	0.09
Pseudo-first order	14.80	0.12	15.60	0.13
Freundlich	-7.01	0.02	-3.13	0.02
Langmuir	16.38	0.04	25.45	0.04

The concentration of atrazine adsorbed at equilibrium in the isolated and mixed treatments ranged from 12.3 to 13.3 (Fig 1A and 1B). However, the presence of glyphosate formulations did not change the concentration adsorbed at equilibrium compared to the treatment alone, p≤0.05 (Table 5). The atrazine adsorption rate (mg kg^-1^ min^-1^) was higher when mixed with the formulations compared to the atrazine alone (Table 5). The highest adsorption rate was observed for the Roundup Ready^®^ formulation (2.3 and 2.0 mg min^-1^) (Table 5). Although not detecting changes in the adsorbed amount at equilibrium, the higher binding speed established between the herbicide atrazine and soil can reduce the time at which the equilibrium is striking. The equilibrium time was estimated between treatments using the pseudo-second order equation. The shortest time to reach equilibrium between atrazine and soil occurred in the treatment mixed with Roundup Ready^®^ (Table 5). The longer time to equilibrium was observed in the atrazine alone (Table 5). The higher adsorption rate capable of reducing the time to reach equilibrium in the mixed treatments is due to higher affinity between adsorbent (soil) and adsorbate (atrazine), probably caused by inert compounds of formulations that promote a higher interaction between herbicide and soil.

**Table 5:** Concentration sorbed at equilibrium (*qe*), rate of adsorption (*k*_*2*_) and estimated time for equilibrium (te) of atrazine in a Red-Yellow Latosol.

Formulations	Glyphosate concentrations					
	10 mg L^-1^			50 mg L^-1^		
	*q*_*e*_	*k*_*2*_	*te*	*q*_*e*_	*k*_*2*_	*te*
Atrazine	12.9 aA	0.8 bA	14.1 aA	12.9 aA	0.9 bA	12.4 aA
Atrazine + ZAPP Qi^®^	12.9 aA	1.3 bA	7.1 bA	12.8 aA	1.4 bA	7.1 bA
Atrazine + Roundup Ready^®^	12.9 aA	2.3 aA	4.3 cA	12.8 aA	2.0 aA	4.9 cA
Atrazine + Roundup WG^®^	13.4 aA	1.0 bA	9.9 bA	13.3 aA	1.0 bA	9.9 bA
Atrazine + Roundup Ultra^®^	12.4 aA	1.0 bA	9.9 bA	12.3 aA	1.0 bA	9.9 bA

Lowercase letters differ *qe*, *k*_*2*_ *and te* among the formulations in each concentration and upper case differ *q*_*e*_, *k*_*2*_ *and te* among concentrations in each formulation by Tukey test at p-value ≤ 0.05. *q*_*e*_ *=* Concentration adsorbed at equilibrium (mg kg^-1^). *k*_*2*_ = second-order constant (mg min^-1^). *te* = Time in equilibrium (min).

Two distinct phases characterized the kinetic model (Fig 1A and 1B). The first moment of the sorption was characterized by rapid sorption, at exponential speed, of the atrazine to the soil. he higher availability of sites for binding on the aggregates' surface allows a more significant interaction with the molecules of atrazine, providing rapid sorption of this herbicide in the soil [22]. The sorption rate of atrazine reduced as a higher amount of atrazine molecules was sorbed to the substrate. The atrazine blind to the soil may elevate the repulsion among molecules presents in the soil solution, reducing the sorption rate [23]. Besides that, after the saturation of the external blind sites, only the sites within the aggregates are available to adsorb the atrazine, and at these locations, the herbicide sorption is slower [24, 25]. A time of 16 hours was adopted in the sorption and desorption tests to ensure the constant concentration of atrazine between soil and soil solution [26].

### Sorption and desorption isotherms

The sorption of atrazine isolated and mixed with glyphosate formulations was determined by testing two commonly used models to understand the adsorption of organic and inorganic compounds in soil [27, 28]. The soil atrazine sorption data were submitted to the Freundlich and Langmuir nonlinear regression models. The two models presented a low standard error for all treatments' parameters, regardless of the dose of glyphosate used (Tables 6 and 7). As in kinetic models, the sorption models were compared for their ability to adjust to the data by the Akaike test, RMSE and R^2^ values. The Akaike test, RMSE and R^2^ values for the Freundlich model were smaller than the Langmuir model (Table 3). Considering these results, the Freundlich isotherm was chosen to understand atrazine sorption in the different treatments.

**Table 6:** Parameters of the Freundlich and Langmuir isotherms for adsorption of atrazine isolated and mixed with formulations of glyphosate (10 mg L^-1^).

Treatment	Freundlich			Langmuir		
	Parameters	Estimated	Std. Error	Parameters	Estimated	Std. Error
Atrazine	*k*_*f*_	2.18 b	0.10	*q*_*max*_	12.90	1.17
	*1/n*	0.73	0.04	*kl*	0.20	0.03
Atrazine + Roundup WG^®^	*k*_*f*_	2.51 a	0.11	*q*_*max*_	22.81	2.93
	*1/n*	0.74	0.04	*kl*	0.11	0.02
Atrazine + ZAPP Qi^®^	*k*_*f*_	2.02 b	0.11	*q*_*max*_	22.51	3.36
	*1/n*	0.82	0.04	*kl*	0.12	0.02
Atrazine + Roundup Ready^®^	*k*_*f*_	1.95 b	0.11	*q*_*max*_	14.73	1.77
	*1/n*	0.79	0.05	*kl*	0.26	0.03
Atrazine + Roundup Ultra^®^	*k*_*f*_	1.91 b	0.11	*q*_*max*_	17.75	2.65
	*1/n*	0.81	0.05	*kl*	0.12	0.03

*k*_*f*_ and *1/n* = Sorption (mg^1-1/n^ kg^-1^ L^1/n^) and linearity constants (dimensionless) of Freundlich model. *q*_*max*_ and *kl* = Maximum concentration sorbed (mg g^-1^) and Langmuir constant (L mg^-1^). Lowercase letters differ in the column the treatments by Tukey test at p-value ≤ 0.05.

**Table 7:** Parameters of the Freundlich and Langmuir isotherms for adsorption of atrazine isolated and mixed with formulations of glyphosate (50 mg L^-1^).

Treatment	Freundlich			Langmuir			
	Parameters	Estimative	Std. Error		Parameters	Estimative	Std. Error
Atrazine	*k*_*f*_	2.18 b	0.05		*q*_*max*_	12.89	1.27
	*1/n*	0.73	0.13		*kl*	0.21	0.03
Atrazine + Roundup WG^®^	*k*_*f*_	2.43 a	0.05		*q*_*max*_	16.76	1.77
	*1/n*	0.77	0.13		*kl*	0.18	0.03
Atrazine + ZAPP Qi^®^	*k*_*f*_	2.00 b	0.06		*q*_*max*_	17.73	2.74
	*1/n*	0.81	0.13		*kl*	0.13	0.03
Atrazine + Roundup Ready^®^	*k*_*f*_	2.09 b	0.05		*q*_*max*_	10.08	0.85
	*1/n*	0.69	0.12		*kl*	0.28	0.04
Atrazine + Roundup Ultra^®^	*k*_*f*_	1.90 b	0.06		*q*_*max*_	17.67	2.86
	*1/n*	0.82	0.13		*kl*	0.12	0.03

*k*_*f*_ and *1/n* = Sorption (mg^1-1/n^ kg^-1^ L^1/n^) and linearity constants (dimensionless) of Freundlich model. *q*_*max*_ and *kl* = Maximum concentration sorbed (mg g^-1^) and Langmuir constant (L mg^-1^). Lowercase letters differ in the column the treatments by Tukey test at p-value ≤ 0.05.

**Fig 3:**
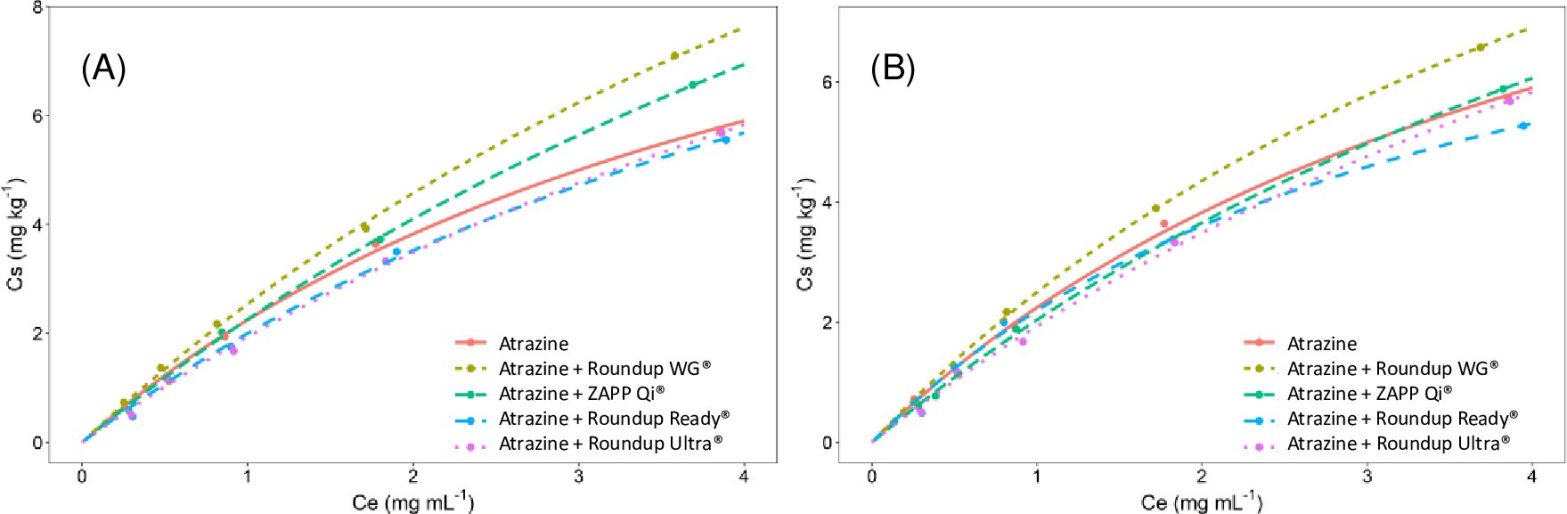
Langmuir isotherms for sorption, in a Red-Yellow Latosol, of atrazine isolated and mixed with glyphosate formulations at concentrations of 10 mg L^-1^ (A) and 50 mg L^-1^ of acid equivalent (B).

The Langmuir model can demonstrate the *q*_*max*_ of one adsorbate to several adsorbents [29]. The *q*_*max*_ variated between 14.73 to 22.51 mg kg^-1^ for treatments isolated and mixed with 10 mg L^-1^ of glyphosate and 10.08 to 17.73 mg kg^-1^ for 50 mg kg^-1^ of glyphosate (Tables 6 and 7). Although this information allows better differentiation between treatments, the estimated values do not belong to the range adsorbed to the soil (0 to 7.82 mg kg^-1^ and 0 to 6.46 mg kg^-1^) measured in the treatments (Fig 3A and 3B). Nonlinear models may estimate values not belonging to the data range in which it was generated [30]. However, estimation errors can occur even for well-adjusted models. The Langmuir model presents a better fit for adsorption tests of heavy metals or other inorganic minerals, such as P, N, and K, where the doses applied to the soil are generally capable of promoting the saturation of the binding sites [31–33]. The doses are very low for herbicides compared to these minerals and seldom saturate the available soil sites. This fact was observed for the adsorption of atrazine isolated and in the mixture, making the Langmuir model less precise to study the behavior of this herbicide in the soil.

The *k*_*f*_ value for the isolated atrazine obtained from the Freundlich isotherm was 2.18 for the concentrations of 10 and 50 mg L^-1^ of glyphosate (Tables 5 and 6). The observed value of *k*_*f*_ in the isolated atrazine test indicates a lower capacity of the substrate to adsorb this herbicide compared to other soil classes already studied. Researches involving a Dystrophic Melanic Gleisol [34], a Volcanic Ultisol [35], and a Purple Latosol [36] showed that these soils adsorb greater amount of atrazine, obtaining in these tests values of *k*_*f*_ equal to 6.09, 15.6, and 4.04 mg kg^-1^ respectively. The high adsorption capacity in these soils was due to their higher cation exchange capacity (14.4, 26.6, 14.1 cmol_c_ kg^-1^). The presence of these negative charges in the soil promotes the adsorption of molecules with positive residual charge, such as atrazine. The atrazine is a weak base, and in soil solution, some molecules can show a positive residual charge that binds at the negative sites of the mineral and organic colloids of soil. The Red-Yellow Latosol used in this study has a low cation exchange capacity (CEC = 3.20 cmol_c_ kg^-1^), and this attribute may promote the low atrazine sorption in the soil.

The *k*_*f*_ of the atrazine on the substrate, when isolated and mixed with ZAPP Qi^®^, Roundup Ready^®^, and Roundup Ultra^®^, did not differ significantly (Tables 6 and 7). The small difference between the *k*_*f*_ values indicate that the presence of these glyphosate formulations does not affect the atrazine sorption process; in other words, the treatments do not alter the amount of this herbicide in the soil solution. Since atrazine sorption is not changed, groundwater contamination's potential does not increase due to the application of the atrazine blend + glyphosate formulations. In areas with high rainfall intensity and soils with high sand content, such as the one used in this work, actions that decrease atrazine sorption can increase the leaching of this herbicide to deeper layers, reaching groundwater sources [37].

The Roundup WG^®^ formulation increased the *k*_*f*_ (11%) compared to isolated atrazine (Tables 6 and 7). The higher *k*_*f*_ indicate that this formulation elevated the atrazine adsorption to the soil compared to the isolated applications of this herbicide. Differently of the others formulation, the Roundup WG® altered the atrazine sorption, evidencing that the presence of inert compounds may change the behavior of atrazine in the soil. The glyphosate in the Roundup WG^®^ formulation presents the lowest molecular weight and ten hydrogen donors and acceptors. The lower molecular weight of glyphosate in the Roundup WG^®^ formulation may allow a higher number of molecules bound to the soil than other formulations. The lower molecular weight of a pesticide has already been associated with higher soil sorption [38]. Therefore, the greater number of glyphosate molecules of the Roundup WG^®^ bound to the soil can raise atrazine sorption via hydrogen bonds, explaining the higher value of Kfs.

Organic and mineral compounds with an adsorption mechanism similar to organic and mineral colloids can compete for the available binding sites, thus reducing the adsorption of those with lower affinity to the sites [39]. This effect has been demonstrated in a study evaluating the azoxystrobin, where the formulation emulsifiable concentrate leached more than suspension concentrate [40]. For glyphosate formulations mixed with atrazine, only Roundup WG^®^ altered the atrazine sorption, demonstrating that the adjuvants and surfactants of the formulated products may have a greater influence on atrazine adsorption than the glyphosate molecule.

Glyphosate is adsorbed to the soil by van der Waals forces, hydrogen bonds, ion exchange (glyphosate may exhibit positive and negative charge varying with pH of the medium) and by covalent bonding with the metallic oxides of the soil, mainly with iron and aluminum oxides (similar to the specific adsorption of inorganic phosphates). This latter is the most important mechanism for oxidic soils [41], as used in this work, causing the glyphosate to remain highly adsorbed in the soil. Unlike glyphosate, atrazine is absorbed primarily by the negative sites and by hydrogen bonds, not competing theoretically for the available binding sites [42]. This fact was evidenced in some treatments in this study, where the atrazine sorption was not altered when mixed with the ZAPP Qi^®^, Roundup Ready^®^ and Roundup Ultra^®^ formulations.

**Fig 4:**
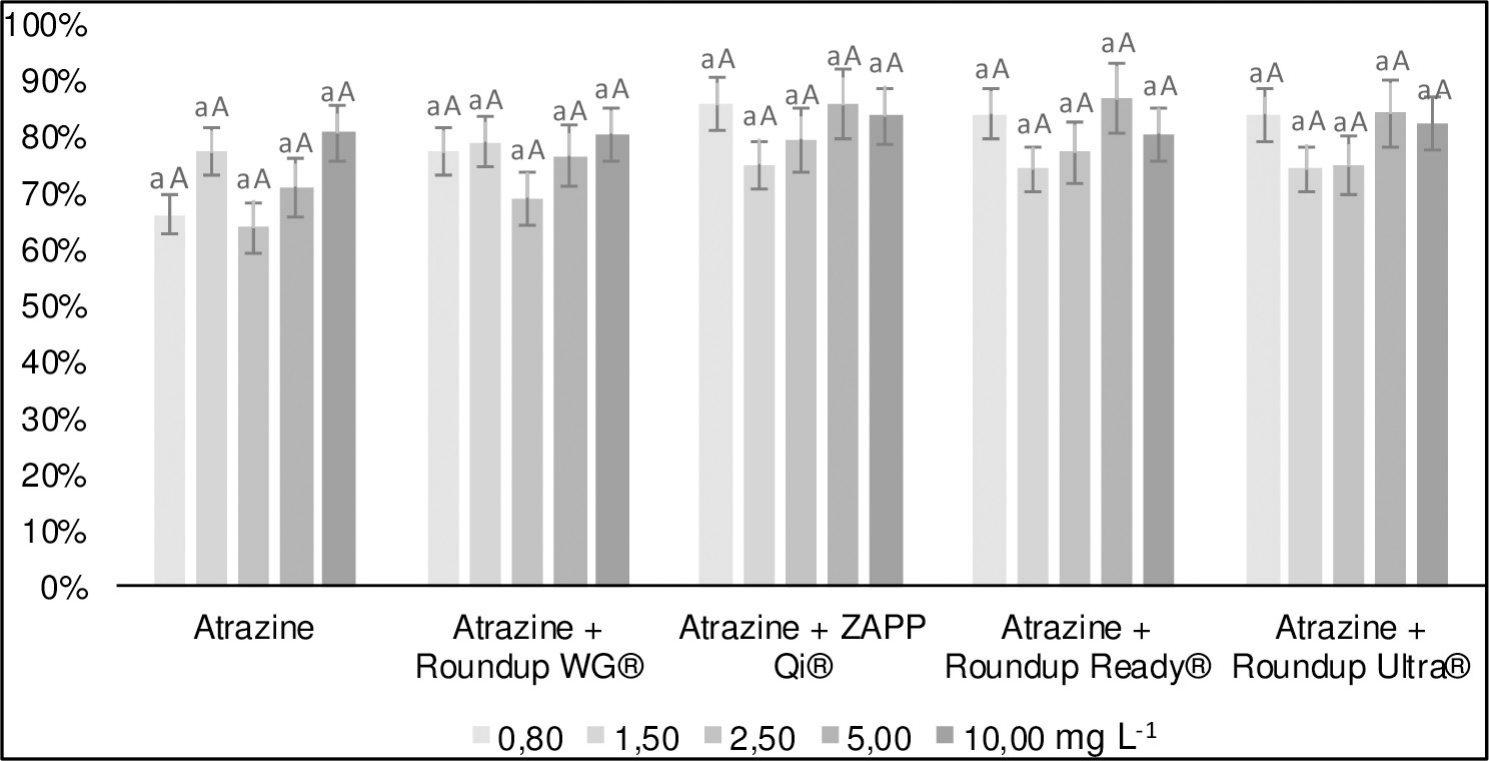
Desorption of atrazine isolated and mixed with glyphosate formulations at concentrations of 10 mg L^-1^ in a Red-Yellow Latosol.

**Fig 5:**
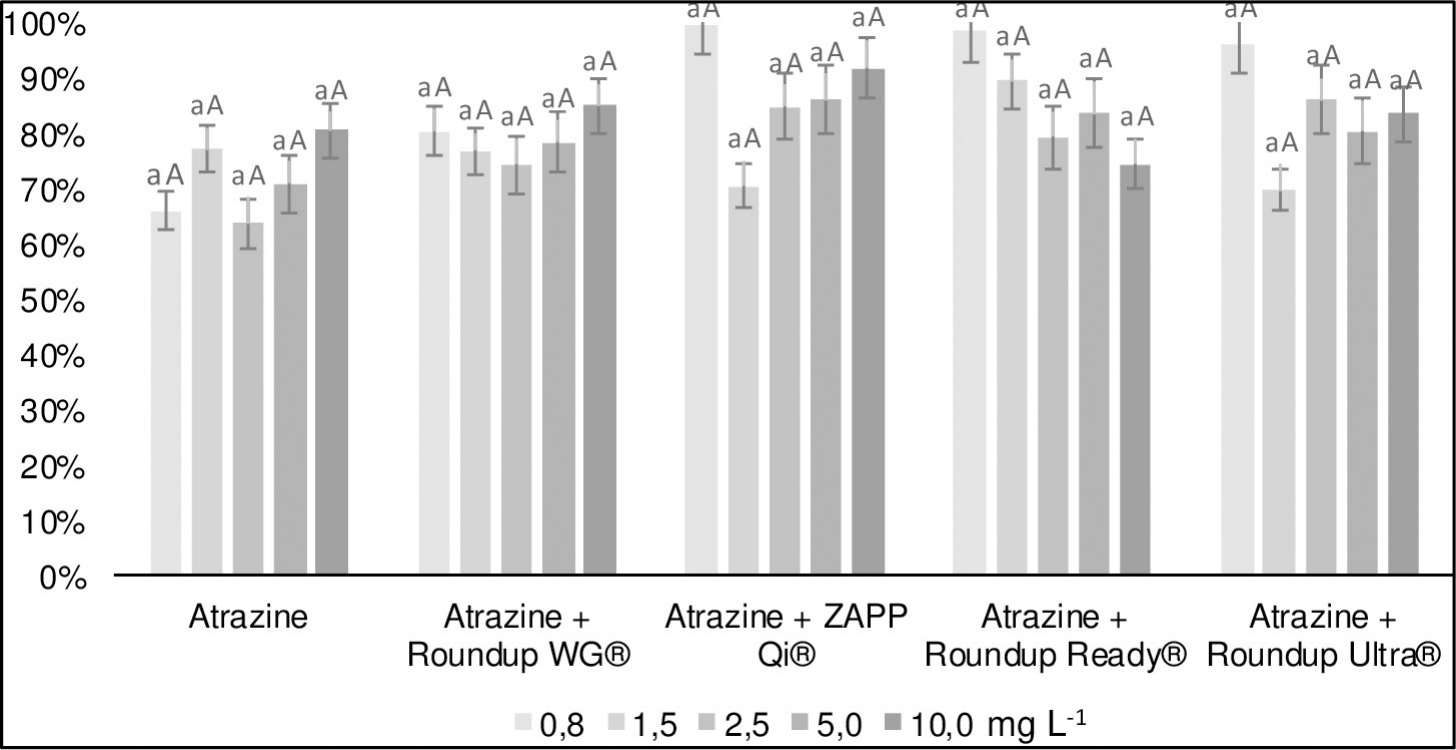
Desorption of atrazine isolated and mixed with glyphosate formulations at concentrations of 50 mg L^-1^ in a Red-Yellow Latosol.

Atrazine desorption was not altered in any of the treatments, regardless of glyphosate concentration, initial concentration formulation of atrazine added (Figs 4 and 5). In all treatments, the atrazine presented high desorption (80%) in relation to the initial amount sorbed to the soil. This phenomenon occurs due to the energy involved in the connections between soil and atrazine. The soil used in work presented low CEC (3.3 cmol_c_ kg^-1^) and clay content (17%). Such properties are unfavorable to atrazine adsorption, mainly due to the polarity between the soil and the herbicide molecule [43]. The high atrazine desorption represents a greater risk of groundwater contamination because it increases the vertical mobility of this pesticide in the soil [37]. For the treatment in mixture with Roundup WG^®^, the high desorption can elevate contamination compared to the other treatments. Greater sorption of atrazine mixed with the Roundup WG^®^ formulation may make the molecule unavailable to be absorbed by the plant or be degraded by the soil microbiota [44, 45].

The results presented in this work show that an herbicide's behavior in the soil can change when in the presence of another molecule. This change occurred differently according to the formulations in which atrazine was mixed. The amount adsorbed at equilibrium was not altered, as observed in the kinetics assays. However, the adsorption velocity of atrazine was altered, with an increase when mixed with Roundup Ready^®^. The sorption capacity of soil for the atrazine also showed a different behavior, and the Roundup WG^®^ elevated atrazine adsorption. These facts emphasize the importance of studies to understand the behavior of herbicides in the soil. The results showed that besides the physical-chemical properties of the soil and herbicide, the mixture of different herbicides molecules or formulations can alter the adsorption mechanisms. It is crucially faced with the facts, that further studies are conducted to identify how these interactions occur and how they can affect environmental quality.

## Conclusion

The adsorption kinetics of atrazine is affected by the presence of glyphosate formulations. The Roundup Ready® formulation reduces the time to the maximum adsorption capacity of atrazine in the soil due to the increase in the adsorption speed. Besides, this formulation does not alter the capacity of atrazine to adsorb to the soil, and consequently, does not increase the risk of leaching or surface runoff of atrazine. In contrast, the atrazine + Roundup Ready® mixture can be safer due to the faster adsorption of atrazine. Atrazine sorption is greater when mixed with Roundup WG® formulation, also indicating less mobility of atrazine in the soil. The other formulations do not alter the atrazine sorption to the soil. No glyphosate formulations change the desorption of atrazine in the tested soil. Our results suggest that the risk of environmental contamination of atrazine when mixed with glyphosate formulations, whether by leaching or surface runoff, is not altered due to not reducing the sorption and increasing the desorption of the molecule.
